# Analysis of Material Handling Safety in Construction Sites and Countermeasures for Effective Enhancement

**DOI:** 10.1155/2015/742084

**Published:** 2015-09-13

**Authors:** C. N. Anil Kumar, M. Sakthivel, R. K. Elangovan, M. Arularasu

**Affiliations:** ^1^Mechanical Department, Regional Centre of Anna University, Coimbatore, India; ^2^Regional Labour Institute, DGFASLI, Government of India, Chennai, India; ^3^Thanthai Periyar Government Institute of Technology, Vellore, India

## Abstract

One of many hazardous workplaces includes the construction sites as they involve several dangerous tasks. Many studies have revealed that material handling equipment is a major cause of accidents at these sites. Though safety measures are being followed and monitored continuously, accident rates are still high as either workers are unaware of hazards or the safety regulations are not being strictly followed. This paper analyses the safety management systems at construction sites through means of questionnaire surveys with employees, specifically referring to safety of material handling equipment. Based on results of the questionnaire surveys, two construction sites were selected for a safety education program targeting worker safety related to material handling equipment. Knowledge levels of the workers were gathered before and after the program and results obtained were subjected to a *t*-test analysis to mark significance level of the conducted safety education program.

## 1. Introduction

Material handling equipment (MHE) is a necessary requirement in construction sites and improper operation of it would result in operator injury or even casualties. Several studies have shown that majority of accidents occur during material transfers involving MHE. Proper safety management systems and procedures for MHE safety should be implemented at the construction sites to prevent these accidents. India's MHE safety rates are good, despite the overall downward trend in recent years, but the situation is still in a serious condition and problems still prevail. Further, human factors also play a vital role in the accidents caused with MHE.

A safety management system provides a systematic way to identify hazards and control risks. Florio et al. [1] expressed that it needs a systematic and comprehensive process to manage safety risks at construction sites. Helander [2] and Neitzel et al. [3] found that about 6% of total building costs are incurred by construction accidents and up to one-third of all fatalities in the construction and maintenance sites are due to the cranes and MHE. Other kinds of accidents that occur in materials handling were studied in [4] using the data gathered from two different sources and the outcome of these studies showed that all accidents reported were during materials transfer process and about 36% of the absenteeism days resulted from these accidents.

Revelle [5] insisted that workers must be educated to identify and report on hazardous conditions and practices. Another study done by Vinodkumar and Bhasi [6], among 1566 employees working in process industrial units, measured the perceptions of employees on self-reported safety knowledge, safety management practices, compliance, and participation in safety activities. In addition to these works, studies have been carried out by several researchers [7–11] on safety with MHE.

In this paper, a safety survey is carried out among sixty construction sites located at different parts of India through a questionnaire survey consisting of twelve elements of safety. The results were then analyzed and taken up for conducting a safety education program at two of the construction sites that scored least among all the other sites. The objective of this program is to develop a knowledge base interface for safety and health management of workers at construction sites. The safety program has been developed with three self-instructional modules which are used to educate nineteen workers each, at site 1 and site 2, respectively. Two questionnaires, one before and one after the program, were used to test the knowledge gained by workers through a *t*-test significance analysis.  Section 3 details the methodology of the survey and safety education program, Section 4 discusses the results, and conclusion is given in Section 5.

## 2. Literature Review

A number of research works and safety training programs have been conducted in several construction industries to inculcate safety in material handling. The construction industries in the EU-27 have provided employment to about 11.5% of the workforce in 2010 while generating an estimated EUR 562 billion of added value [12]. And in 1998, the largest number of workplace fatalities has been reported, compared to any other industry accounting for almost 20% of the total deaths [13–15].

A quantified risk estimation technique was analysed and applied on construction worksites through proposed solutions to degrade the likelihood of arising fatal accidents from the outcome of risk value [16]. Fung et al. [17] explored 14 common types of trades, accidents, and accident causes and investigated people's need involved in construction to take systematic and effective risk assessments for different trades through a developed risk assessment model. Zwetsloot et al. [18] analyzed the risk control of four certification and testing regimes (CTRs) in the Netherlands to create an understanding of problems arising in risk control through such regimes and identify critical processes and factors that can affect those risk control process.

Recent studies show evidences that health and safety training have made a difference in the safety knowledge of construction workers [19]. A questionnaire was designed and applied to 40 construction workers in southern Spain to study the impact of health and safety investment on construction company costs [20] and the same was discussed with slight alterations by El-Mashaleh et al. [21] for Jordanian construction industry. Park and Kim [22] in their research work proposed a framework and a prototype system for a novel safety management and visualization system (SMVS) including four other construction technologies, building information modeling (BIM), location tracking, augmented reality (AR), and game technologies. A virtual fencing technology was proposed by Williams et al. [23] that triggers warning alerts to prevent workers from standing in hazardous positions.

In addition to research works related to MHE safety, articles have also been published considering other factors affecting worker safety such as in [24] whose work undertakes a comparative study of HR practices adopted for safety management on construction projects in the United States (US) and Singapore. Another research method was proposed by Teoa and Ling [25] using a method consisting of 15 steps including a survey to develop and test safety management system audit tools being used to assess the effectiveness of construction site's safety system. Also, safety management system's implementation and development have been discussed in [26–32].

Shepherd et al. [33] estimated that the crane accidents are the reason for about 25–33% of casualties in construction activities. A checklist of necessary safety precautions being followed in Saudi Arabian construction sites was surveyed by Jannadi and Assaf [34]. According to Häkkinen [35, 36], the nature of lifting work depends on how the crane is being operated. One method to prevent accidents is to mitigate the possibilities of disturbances by maintaining the work environment suitable to physiological and psychological demands of human.

## 3. Description: Material Handling Safety Analysis

Implementation and strengthening of the safety management of MHE are a tough task and MHE being used in construction sites requires continuous and extensive studies to improve its operation safety. Construction sites are classified as three types: small scale, medium scale, and large scale. Each of these types has a unique safety management system peculiar to its own construction sites.

The first part of this research work has been carried out considering two segments of the construction sites: small and medium scale sites. Construction sites employing less than 50 workers are termed small scale and those which employ between 50 and 100 workers are termed medium scale construction sites. Since most of the large scale sites follow all necessary safety procedures, they are not considered for this study.

### 3.1. Elements of Safety

For this study, the MHE safety management system of small and medium scale sites has been divided into twelve separate elements constituted in two parts: administrative management system and technical management system. The elements of safety were derived from the literature and general risk assessments in construction sites. Each of the elements had a questionnaire in its related field with ten questions for each element totaling to 120 questions overall. The first six elements come under safety management system which include general working standards, health and hygiene, personal protective equipment, hazard and risk identification, inspection of cranes, and worker behavioral safety and the other six elements come under technical management system which are tower crane and hydra crane inspection, operation of industrial trucks and dumpers, safety of passenger and builder hoist, safety during storage and material handling, safety of earth moving equipment, and prevention of fire and fire protection.

The questionnaire was sent to thirty small and medium scale construction sites at different parts of India out of which twenty-eight sites from small scale and twenty-nine sites from medium scale sites answered for the questionnaires. One set of answers from the medium scale site have been neglected due to irrelevance in answers. The questions were a “yes” or “no” type and the number of questions answered “yes” was counted and considered for this analysis. Tables 1 and 4 show the total points obtained by small and medium scale sites, respectively.

The values of mean and correlation coefficient are found out as shown in the following example.

Consider site 1 shown in Table 1. The split-up of points obtained in each of the twelve elements is shown in Table 2. The value of 53 is the sum of all the “yes” for each module and the mean of points obtained in site 1 is (3 + 5 + 5 + 3 + 3 + 4 + 6 + 4 + 5 + 5 + 5 + 5)/12 = 53.

The correlation coefficient “*r*” is calculated using (1) for which the preliminary data is calculated as shown in Table 3. The summation values of Table 3 are applied in the equation to find out “*r*” as given in (2). The final value of “*r*” for site 1 is obtained as −0.64; similarly “*r*” is calculated for the rest of the sites:

(1) r=nΣxy−ΣxΣynΣx2−Σx2nΣy2−Σy2,

(2) r=6113−23306∗93−2326∗152−302.

### 3.2. Safety Education Program for MHE

The implementation of safety education program was done through a method of acclaimed safety procedures, in which the workers were given self-instructional modules containing materials for the education program.

Based on the analysis of our study, the knowledge level of safety among workers is less than required. This can be seen with the mean values and mostly negative correlation coefficient as shown in Tables 1 and 4. Hence, for the purpose of educating the workers on safety with MHE, three modules considered to be closely relevant for the effective improvement of worker safety were developed. The modules are general worker safety, inspection and operation of MHE, and safety of MHE environment.

These three modules of safety were considered to be effective for the education program and typical for enhancing the awareness on safety among industrial workers. Two construction sites, one from small scale and the other from medium scale, which received the lowest score were taken up and about nineteen workers from each of the construction sites were selected for the education program. The objective of the education program is to inculcate knowledge of safety to the workers while they work with the MHE.

### 3.3. Effectiveness of the Program

A different set of questionnaire was once again issued to the workers before and after the program to find out its effectiveness. The questionnaire consisted of three parts: general worker safety, inspection and operation of MHE, and safety of MHE environment with 40 questions for each part totaling to 120 questions and each “yes” contributes to 0.25 points. Then, the points acquired by each worker before and after the test were compared to find out the improvement in the knowledge level of the workers. The mean, standard deviation, and correlation coefficient of the results of both the questionnaire sets before and after the program were calculated and the significance value was found out using a two tailed *t*-test analysis. Results of the points obtained by the workers for the questionnaire before the program are shown in Table 5. It shows the points obtained by workers before training.

## 4. Results and Discussions

In the analysis of safety management system for each of the twenty-eight small scale and medium scale construction industries, respectively, the data received in response to the questionnaire were tabulated and analyzed for mean, standard deviation, and correlation coefficient.

Subsequently, the education program for workers that was conducted to improve their safety knowledge resulted in effective improvement of the worker's safety awareness. They were marked against a maximum of 40 points per module accounting to 120 as overall maximum points for all three modules together. The significance analysis results for the education program are showed in Table 6.

On appraising the outcomes of two sites before and after the safety training program on MHE, the results were appealing and better than the previous work in [37]. Contrary to the previous work, the training modules were prepared in such a way to focus on improving worker's safety culture and their knowledge in engineering and administrative and management control, explaining more on accident prevention. The modules continuously stressed a fact that personal protective equipment should be the last resort for safety as it only protects after an incident has occurred but does not prevent the incident from happening. The questionnaire was also developed in same manner to assess the workers and answers showed improvements in points obtained by workers after the program. The positive correlation coefficient values proved knowledge improvement among the workers of both sites. Also, the two tailed *t*-test showed that the safety knowledge of workers was above the critical value of 2.04 at about 0.05 significance level for both construction sites.

**Figure 1:**
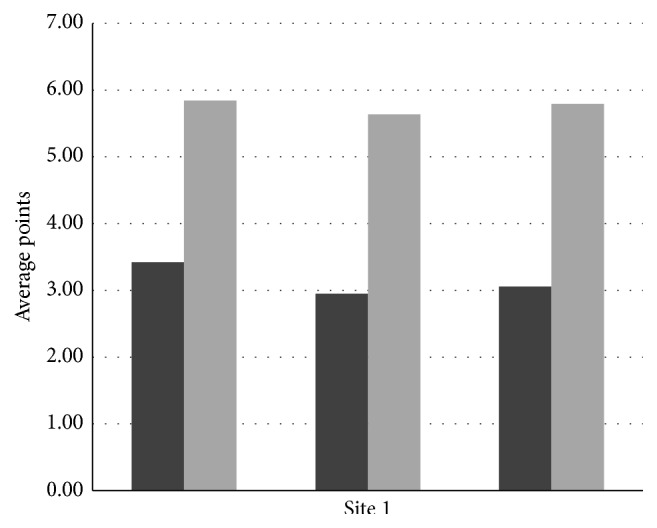
Graph showing effectiveness of the program at site 1.

**Figure 2:**
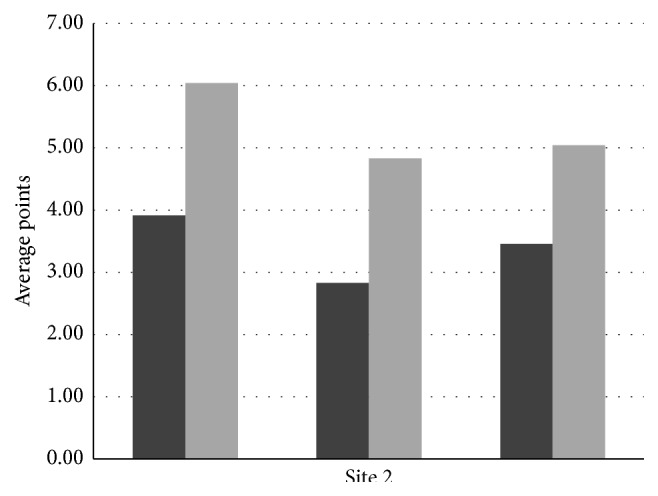
Graph showing effectiveness of the program at site 2.

The mean points obtained before and after the tests by workers of site 1 are 7.46 and 13.61 and those of site 2 are 10.21 and 15.92, respectively. Similarly, the standard deviation values before and after the tests at site 1 are 2.45 and 1.85, respectively. Likewise, the standard deviation values for site 2 are 2.62 and 1.85. Correlation coefficient and *t*-test values for site 1 are 0.53 and 8.79 and those for site 2 are 0.56 and 7.74, respectively, for a 0.05 significance level. The graph in Figures 1 and 2 shows the effectiveness of the three modules of safety at the two construction sites. The average points of site 2 are slightly higher than those of site 1 while the percentage increase is almost the same for both sites. Also the standard deviation has decreased for both sites after the program, while the *t*-test significance value has increased very well indicating the potential of the safety education program.

## 5. Conclusion

The safety education program for MHE workers was carried out with an aim to improve their self-awareness and change the cultural mindset to mitigate all actions that will lead to harm and educate them more on preventative control methods. And the program was successful in improving safety awareness and culture by educating the workers at the two construction sites. The modules focused more on engineering and administrative and management control, training the workers to avoid hazards rather than relying on personal protective equipment for their protection. Although the self-instructional modules were used for worker's educational purposes, the program's success was solely dependent on their motivation to learn. Another was the lack of management commitment towards educating their workers about safety. In the future, the safety education program is to be extended with additional modules covering other unsafe areas at the construction sites and to inculcate commitment among management towards safety.

## Figures and Tables

**Table 1:** Points obtained by small scale construction workers.

Small scale construction sites			
Site	Total (max. = 120)	Mean	Correlation coefficient
1	53	4.42	−0.64
2	61	5.08	0.00
3	38	3.17	−0.75
4	53	4.42	0.33
5	55	4.58	−0.60
6	50	4.17	0.81
7	58	4.83	−0.56
8	56	4.67	−0.18
9	54	4.50	−0.12
10	54	4.50	−0.19
11	58	4.83	−0.67
12	59	4.92	−0.16
13	58	4.83	−0.81
14	60	5.00	−0.29
15	61	5.08	−0.94
16	51	4.25	−0.63
17	52	4.33	−0.40
18	67	5.58	−0.51
19	55	4.58	0.45
20	55	4.58	−0.26
21	54	4.50	−0.05
22	67	5.58	−0.52
23	60	5.00	−0.24
24	62	5.17	0.24
25	63	5.25	0.21
26	53	4.42	0.66
27	61	5.08	−0.45
28	38	3.17	0.67

**Table 2:** Element wise points for site 1.

Site 1	Safety modules												Total
	Administrative management system						Technical management system						
	1	2	3	4	5	6	7	8	9	10	11	12	
Points	3	5	5	3	3	4	6	4	5	5	5	5	53

**Table 3:** Calculation for correlation coefficient.

Element	*X*	*Y*	*X∗Y*	*X* ^2^	*Y* ^2^
1	3	6	18	9	36
2	5	4	20	25	16
3	5	5	25	25	25
4	3	5	15	9	25
5	3	5	15	9	25
6	4	5	20	16	25
*∑*	23	30	113	93	152

**Table 4:** Points obtained by medium scale construction.

Medium scale construction sites			
Site	Total (max. = 120)	Mean	Correlation coefficient
1	58	4.83	0.56
2	48	4.00	−0.22
3	50	4.17	−0.14
4	47	3.92	−0.59
5	51	4.25	0.33
6	52	4.33	−0.06
7	53	4.42	0.03
8	41	3.42	0.69
9	43	3.58	−0.09
10	51	4.25	0.00
11	53	4.42	−0.39
12	50	4.17	−0.33
13	51	4.25	0.05
14	53	4.42	−0.60
15	60	5.00	−0.24
16	55	4.58	0.18
17	49	4.08	−0.74
18	47	3.92	−0.47
19	51	4.25	−0.13
20	50	4.17	0.19
21	68	5.67	−0.06
22	54	4.50	−0.35
23	54	4.50	−0.45
24	52	4.33	0.56
25	54	4.50	−0.37
26	59	4.92	−0.26
27	51	4.25	−0.19
28	57	4.75	0.42

**Table 5:** Points obtained by workers before training.

Sl. number	General worker safety (max. = 10)		Inspection and operation of MHE (max. = 10)		Safety of MHE environment (max. = 10)		Total of all areas of safety (max. = 30)	
	Site 1	Site 2	Site 1	Site 2	Site 1	Site 2	Site 1	Site 2
1	4	4	3	2	4	4	11	10
2	2	3	4	3	3	5	9	11
3	1	5	5	1	5	3	11	9
4	3	3	3	2	2	4	8	9
5	4	4	2	4	4	5	10	13
6	3	3	4	3	6	3	13	9
7	5	7	5	5	4	4	14	16
8	7	6	3	4	3	5	13	15
9	3	5	1	6	5	4	9	15
10	2	5	2	4	2	6	6	15
11	3	7	5	3	1	4	9	14
12	1	4	2	1	2	7	5	12
13	5	6	3	4	3	2	11	12
14	3	5	1	3	2	3	6	11
15	6	6	0	6	4	5	10	17
16	4	5	3	5	2	3	9	13
17	2	3	2	4	3	5	7	12
18	5	6	4	5	1	5	10	16
19	2	7	4	3	2	6	8	16

**Table 6:** Results analysis of the safety education program.

Site 1	Mean	Before training	7.46
		After training	13.61
	Standard deviation	Before training	2.45
		After training	1.85
	Correlation coefficient	0.53	
	*t*-test value	8.79	

Site 2	Mean	Before training	10.21
		After training	15.92
	Standard deviation	Before training	2.62
		After training	1.85
	Correlation coefficient	0.56	
	*t*-test value	7.74	
